# An unusual pigmented skin lesion on the nipple‐areola area

**DOI:** 10.1002/ccr3.7115

**Published:** 2023-04-12

**Authors:** Kouki Chaima, Rim Chaabouni, Emna Bahloul, Sonia Boudaya, Mariem Amouri, Hamida Turki

**Affiliations:** ^1^ Department of Dermatology Hedi Chaker Hospital Sfax Tunisia

**Keywords:** nipple dermatosis, pigmentary disorders, terra firma‐forme dermatosis

## Abstract

Considering the rarity and underdiagnoses of this disorder, a pigmented and hyperkeratotic skin lesion located on the trunk, resembling to acanthosis nigricans should always be investigated for terra firma‐forme dermatosis (TFFD) and thus alcohol must be applied. TFFD should be known among dermatologists and can be easily diagnosed and treated with isopropyl alcohol.

A 17‐year‐old woman presented to our department with a 2 year history of hyperkeratotic papules affecting her areolae and nipple. Her medical history was unremarkable. The patient reported the disappearance of the crusts erupt over time even without therapy. Cutaneous examination revealed symmetrical brownish and hyperkeratotic papules on the nipple‐areola area (Figure 1).

**FIGURE 1:**
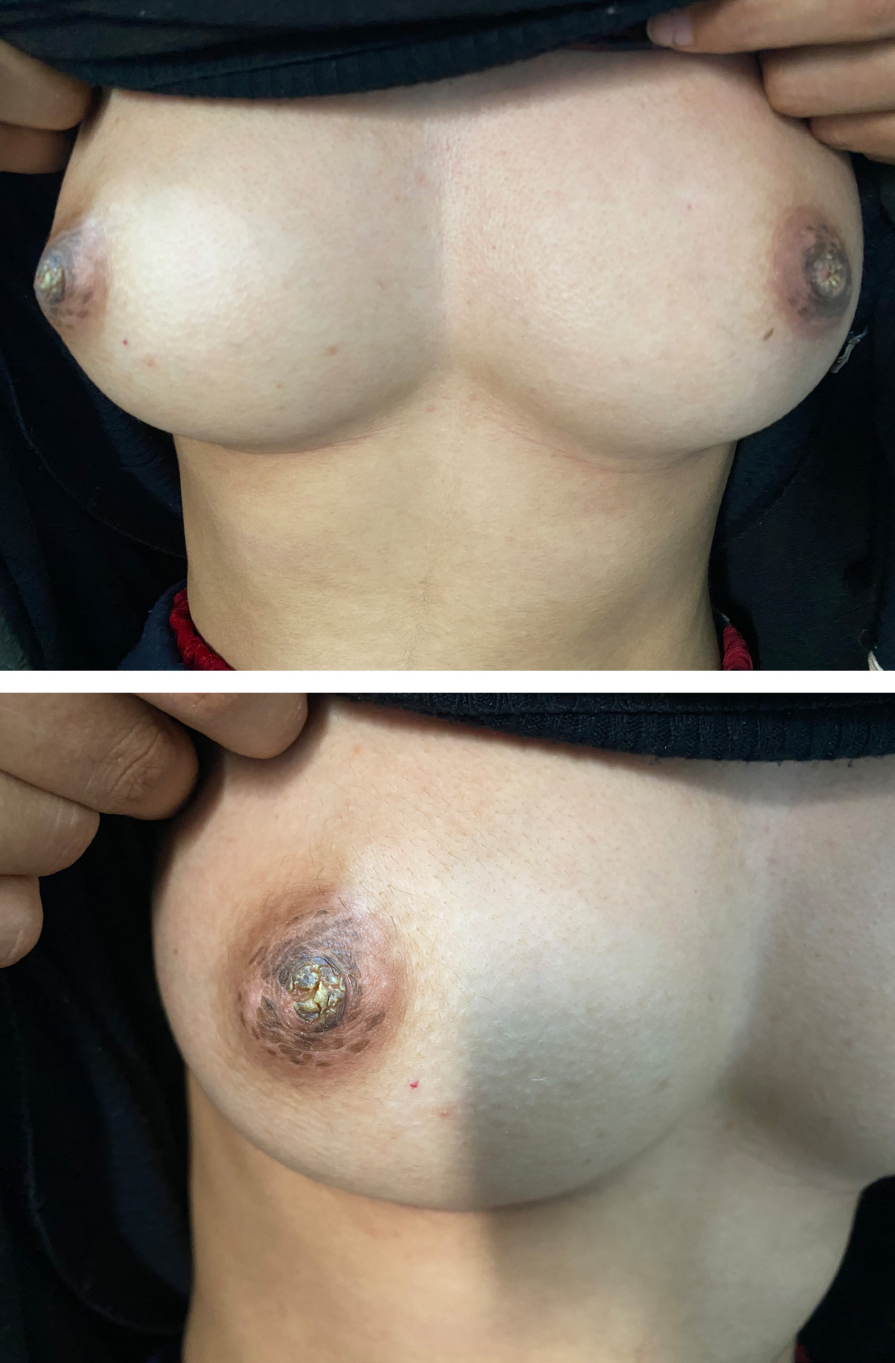
Symmetrical brownish and hyperkeratotic papules on the nipple‐areola area.

## What's your diagnosis?

**FIGURE 2:**
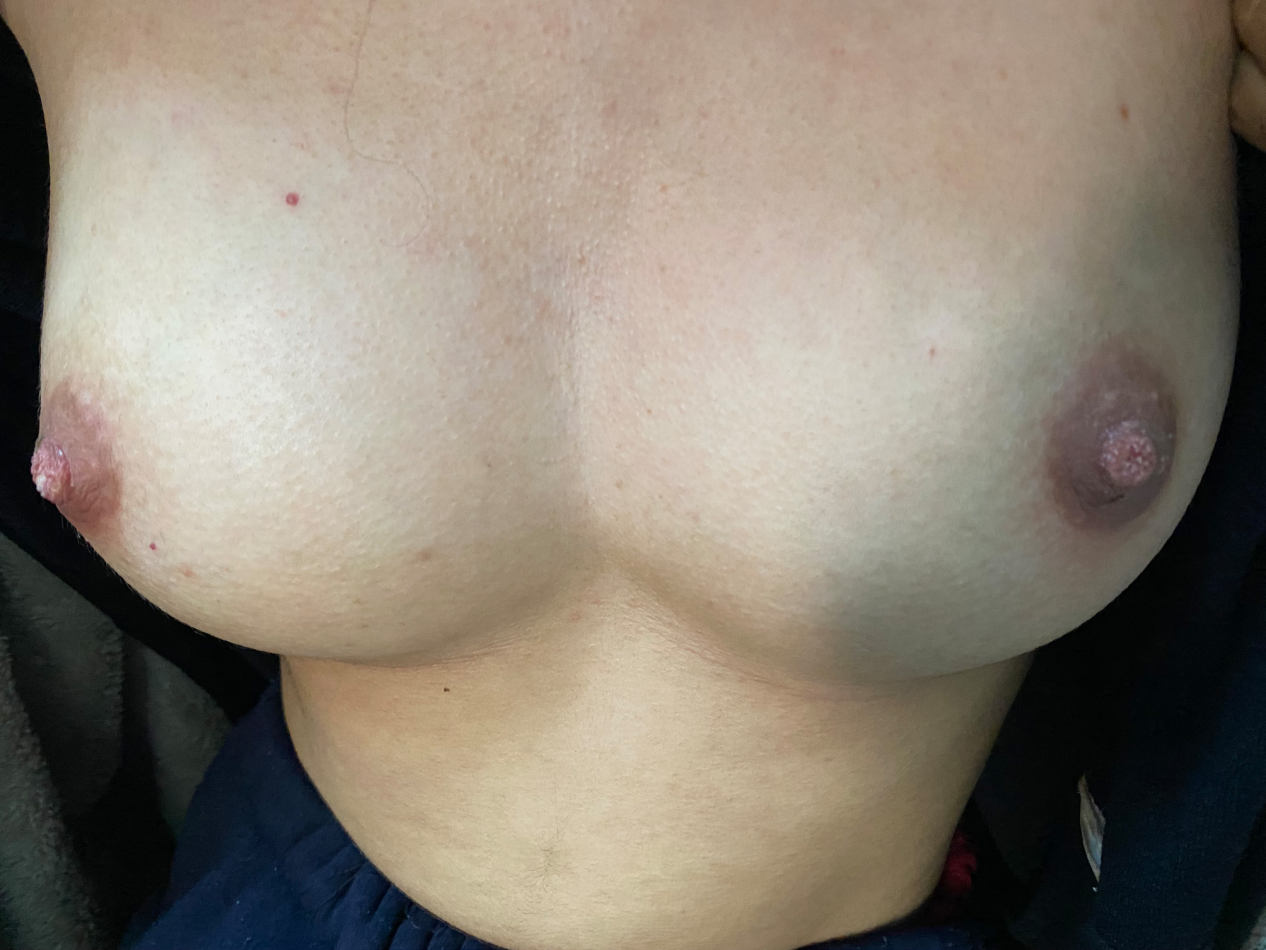
A complete resolution of all the lesions after the application of 70% isopropyl alcohol.

Given the clinical presentation, we considered terra firma‐forme dermatosis (TFFD), hyperkeratosis nevoid of the nipple, dermatosis papulosa nigra, or an atypical form of acanthosis nigricans. We observed a complete resolution of all the lesions after the application of 70% isopropyl alcohol, (Figure 2) which confirmed the diagnosis of TFFD. The patient was advised to apply a topical keratotic cream or isopropyl alcohol in case of recurrence of the skin eruption.

TFFD is considered as an underdiagnosed acquired pigmentation disorder.^1^ Its exact etiology remains unclear. A highest prevalence was previously observed in the pediatric population.^1^, ^2^ Clinically, it represents as multiple brownish patches or hyperkeratotic plaques resistant to washing with common soap and typically removed with 70% isopropyl alcohol. This finding, known as “alcohol wipe test,” is pathognomonic for this dermatosis. It is especially observed in the face, neck, and trunk. Only few cases of TFFD of the nipple have been reported.^2^ The disease is commonly considered as benign, but cosmetic distress in affected patients can occur. Differential diagnosis encompasses: dermatosis neglecta, acanthosis nigricans, tinea versicolor, confluent and reticulated papillomatosis, seborrheic keratosis, and epidermal nevi. Even if they resemble TFFD, they cannot be removed by using rubbing alcohol.^2^

TFFD should be known among dermatologists in all cases of reticulate hyperpigmentation of the trunk and the extremities, particularly in children.

## AUTHOR CONTRIBUTIONS

**Kouki Chaima:** Conceptualization; investigation; validation; visualization; writing – original draft; writing – review and editing. **Rym Chaabouni:** Conceptualization; data curation; writing – review and editing. **Emna Bahloul:** Methodology; visualization; writing – original draft; writing – review and editing. **Sonia Boudaya:** Supervision; validation. **Mariem Amouri:** Supervision; validation; visualization. **Hamida Turki:** Project administration; supervision; writing – review and editing.

## FUNDING INFORMATION

We received no funding to support for this work.

## CONFLICT OF INTEREST STATEMENT

We have no conflict of interest to disclose concerning this work.

## CONSENT

The patient in this manuscript has given written informed consent to the publication of the case details. Written informed consent was obtained from the patient to publish this report in accordance with the journal's patient consent policy.

## Data Availability

Not available.
